# Supplementary material for: Efficacy of Guardian Cap Soft-Shell Padding on Head Impact Kinematics in American Football: Pilot Findings
Source: Int J Environ Res Public Health. 2023 Oct 28;20(21):6991. doi: 10.3390/ijerph20216991 (PMC10650906; doi:10.3390/ijerph20216991)
Supplement: Supplementary file 1 [file ijerph-20-06991-s001.zip › ijerph-2624988-supplementary.pdf]

Table S1. CHAMP 2022 checklist of information to include when reporting studies of on-field deployment and validation of wearable head acceleration measurement devices.

Checklist item	Explanation	Example(s)	Reported on Page No
1. Sensor Technology and Specifications			
(1a) Device model name	The name or model of device used to collect data	PMID: 15654184, ''This study used the Head Impact Telemetry (HIT) System (Simbex, Lebanon, NH)…consists of sensors (6 linear accelerometers and 1 temperature)…''	3
(1b) Sensor type	The type of sensor (e.g., triaxial linear accelerometer, triaxial ARS)	PMID: 339862230, ''The SIM-G device comprises a high and low-g triaxial accelerometer for linear acceleration measurement (3 g–150 g) and a triaxial gyroscope for angular velocity measurement''	3
(1c) Sensor sample rate	The sampling rate of the sensor	PMID: 29809079, ''The sensor sampled linear accelerations at a rate of 1 kHz and rotational accelerations at 800 Hz.''	3
(1d) Sensor magnitude range	The range of magnitudes the sensor can record	PMID: 339862230, ''The SIM-G device comprises a high and low-g triaxial accelerometer for linear acceleration measurement (3 g–150 g) and a triaxial gyroscope for angular velocity measurement''	3
(1e) If applicable, device hardware/firmware version number	The version number related to the hardware/firmware for the device	PMID: 29373056, ''The Smart Impact Monitor (SIM; firmware version 3.7; SIM-G, version 3.3; AP, version 0.9.150413; software, Triax Technologies, Norwalk, CT) was used to quantify head acceleration.''	3
(1f) Recording trigger threshold	The sensing threshold (e.g., 10 g) for an event to be recorded on the head acceleration measurement device and how the trigger threshold is evaluated	PMID: 32913379, ''The pre-set trigger for the device to record and download an impact was 10 g. Any impacts below this threshold were not recorded.'' PMID: 31388849, ''Data acquisition triggered any time a single accelerometer exceeded a 9.6-g threshold.''	3
(1g) Pre- and post-trigger duration	Duration of pre-trigger data recorded	PMID: 27598519, ''When an impact above the threshold occurred, information regarding 10 ms before and 52 ms after the impact was transmitted…''	3
(1h) Device form factor and attachment	The type of device/how the device is mounted (e.g., mouthguard)	PMID: 33152691, ''Each athlete was fit with a custom-fit mouthpiece instrumented to measure linear and rotational head kinematics during on-field impacts,''	3
(1i) If applicable, device fitting procedures	Procedures for fitting wearable device (e.g., custom-formed mouthguard)	PMID: 33152691, ''Dental impressions were obtained from each athlete by a trained dental professional, and a dental model was poured from the dental impression. A custom-fit mouthpiece made of acrylic material was created for each athlete.''	3
(1j) If applicable, helmet manufacturer/model name	The name of the manufacturer/model of the helmet worn by participants in helmeted sports	PMID: 32936594, ''Eligible participants for the study wore a Riddell Revolution, Speed, or Speed Flex helmet to accommodate a HIT System encoder…''	3
(1k) Evidence of device kinematic validation	Details regarding device kinematic validation that exists in prior literature	PMID: 29321637, ''The mouthguard…has approximately 10% error in measuring peak head linear acceleration, angular acceleration, and angular velocity in dummy head validation (ref).''	3

Checklist item	Explanation	Example(s)	Reported on Page No
2. On-Field Logistics			
(2a) Participants	Eligibility criteria for participants (e.g., sport, sex/gender, age, level of play), number of participants approached, number of participants enrolled, number of participants that were ultimately excluded (e.g. incomplete data sets, dropped out of study)	PMID: 31240507, "A total of 340 players from six National Collegiate Athletic Association (NCAA) football programs (two of which were military service academies) participated in this study…"	3
(2b) Study settings/location	Settings and locations where the data were collected (e.g., laboratory, field, practice, games, tournaments)	PMID: 30362082, "Head impact data were recorded for all practice, scrimmage and game activities during the 2015, 2016, and 2017 football seasons, including spring practice, preseason training camp, and regular season practice and games."	3
(2c) Data collection dates	Seasons/years the data were collected	PMID: 30362082, "Head impact data were recorded for all practice, scrimmage and game activities during the 2015, 2016, and 2017 football seasons, including spring practice, preseason training camp, and regular season practice and games."	3
(2d) Device usage	Actions to verify device usage (e.g., device is functional, battery is charged, device is attached securely and properly)	PMID: 32255667, "All X-Patch devices were tested for basic functionality (eg, battery life) before use…86 players had patches that detached (60%) or became faulty (40%), and HAEs from these patches were excluded from further analysis."	3
3. Head Acceleration Event Verification			
(3a) Method of impact verification	Method used to confirm device-recorded events were actual head acceleration events (e.g., video or observer, support vector machine classification, proximity sensor); state if none used	PMID: 33738313, "Video review of all sensor-recorded events was used to identify actual head impact events…" PMID: 34549342, "All recorded events were processed by the MiGNet program, a validated, deep learning algorithm that distinguishes true head impacts from false positive events caused by mouthguard handling, application, or other movements that are unrelated to impacts (ref)." PMID: 32130020, "…the proprietary manufacturer software labeled each sensor-recorded event as either a "valid" or "spurious" impact."	3
(3b) Time synchronization method	Methods used to synchronize video and wearable device clocks (e.g., video recording the machine clock)	PMID: 28541813, "At the start of each game, with the game official's audible whistle and the start of the game clock, the videographer displayed a visual marker (clock) of the date and start time of each game. Simultaneously, the "dummy" sensor was struck 5 times in full view of the camera, with the first impact being used to synchronize. At the conclusion of each game, the same procedure was repeated to time stamp the end of play."	3
4. Data Windowing			
(4a) Temporal windowing	Parameters and methods used for time-windowing (e.g., start and end timepoints of session, elimination of scheduled and unscheduled stoppages, based on video confirmation)	PMID: 32130020, "…the timestamp in the video footage was used to determine the time points associated with the start and end of each half, as indicated by the whistle of the referee, and sensor data outside of verified game times were excluded."	3

Checklist item	Explanation	Example(s)	Reported on Page No
(4b) Temporospatial windowing	Parameters and methods used for player-windowing (e.g., timepoints when a positional group or player enters or leaves the playing area during the session, start and end timepoints of an athlete actively participating in the session, based on video confirmation, proximity sensors, global positioning system)	PMID: 26674407, "All accelerations recorded via the helmets were crosschecked to the Team AMS software [GPS data] to investigate when and where the event occurred."	3
(4c) Kinematic windowing	Parameters and methods used for windowing the events based on kinematic measures	PMID: 31388849, "Any impacts with peak resultant linear acceleration below 10 g were not included in this analysis as they can be associated with non-impact dynamic movements in the athlete… System output files to ensure that all athlete and impact information values were included, filtering out any impacts that exceeded 200 g and 10,000 rad/s2"	
5. Video Verification			
(5a) Type of video review, if applicable	Guided video review (e.g., to confirm true positive events and identify/remove false positive events) and/or blinded video review (e.g., to quantify false negatives)	PMID: 33078368, "In the first stage, a pool of 16 human reviewers were blinded to the sensor event data and identified head impacts occurring to instrumented players on the field (1 reviewer per player)."	4
(5b) Video recording parameters, if applicable	The number and type of video recording devices (e.g., number of cameras, placement, resolution, and frame rate of video recordings)	PMID: 27432843, "A research assistant captured game video by using a professional grade video camera (Panasonic HMC-40, Secaucus, NJ) placed above the press box ~3 stories high at the 50-yard line. Video was recorded in full high-definition with a resolution of 1080 $\times$ 720 at 24 frames per second."	3
(5c) Video/observer review parameters, if applicable	The number and type of video reviewers or on-field observers (e.g., number of raters, calculations of inter- and/or intra-rater reliability, level of experience)	PMID: 31075762, "Two independent reviewers analyzed the on-field video data using Kinovea (experimental version 0.8.26) video analysis software independent of the biomechanics."	3
(5d) Contextual information of head impacts	Other head acceleration or head impact event parameters characterized from video/observer (e.g., impact location)	PMID: 32303477, "…the videos were later reviewed by research staff to eliminate false positives, confirm impact locations on the head, and identify impact mechanisms and player positions."	3
6. Advanced Post-processing			
(6a) Data transformation	Methods used to transform recorded data to analyzable data (e.g., numerical integration from angular velocity to angular acceleration, transformation from the location of the sensor to the center of gravity of the head, must disclose if a "black box" algorithm was used)	PMID: 34689676, "…mouthpiece-recorded data were filtered, zero-offset, rotated to match a conventional coordinate system, and transformed to the athlete's head centre of gravity using a subject-specific transformation."	NR
(6b) Kinematic data filtering	Any filtering used for processing data collected from a sensor; must disclose if manufacturer "black box" post-processing was used. Include offset removal	PMID: 34463209, "All data were filtered using a 4th order, zero lag, low-pass Butterworth filter to remove high-frequency noise. A single cut-off frequency was not found to be appropriate for all impacts, due to variability in the underlying signal components. Consequently, impact-specific, optimal cut-off frequencies were determined for each impact using residual analysis. Filtering was applied to vector component data."	3

Checklist item	Explanation	Example(s)	Reported on Page No
(6c) Other post-processing techniques	Any software or hardware used for processing data collected from a sensor (e.g., impact detection filtering, infrared system); must disclose if manufacturer "black box" post-processing was used. Provide details on validation of post-processing techniques (e.g., training data set used)	PMID: 29321637, "A two-class SVM [support vector machine] classifier was trained to differentiate head impacts from the nonimpacts." PMID: 29321637, "Prior to training the classifier, we used infrared (IR) device placement measurements to filter out recordings where the mouthguard was not coupled to the upper jaw. Then, features were extracted from the kinematic sensor measurements to train an SVM classifier that distinguishes between impacts and nonimpacts."	3
7. Data Reporting			
(7a) Event definition	A head acceleration event is defined as an event/incident that gives rise to an acceleration response of the head caused by an external short-duration collision force applied directly to the head or indirectly via the body in sport, recreational, military, or other activities of interest (e.g., direct or indirect head acceleration events); a head impact event is defined as a contact event involving direct contact to the head (i.e., direct head acceleration event)	PMID: 33183139, "Head acceleration events (HAEs) were monitored using the xPatch."	3
(7b) Device-recorded events	Number of events reported by the devices before verification and/or processing	PMID: 30802147, "…the MV1 sensors recorded 2039 nominal head impact events…"	3
(7c) Head acceleration events	Number of true positive acceleration events after verification and post-processing	PMID: 33986230, "Female athletes sustained 271 head impacts during 18 games and male athletes sustained 1041 head impacts during 23 games."	3
(7d) Device performance	False positive rates (and/or false negative rates with blinded review)	PMID: 31000457, "Among the 66 (56.1%) cases with HAEs (PLA ≥ 30 g) that could be verified on video, 48 (72.7%) were true positive for direct or indirect head impact and 18 (27.3%) were false negatives."	3
(7e) Athlete exposures	If reporting rates, definition of athlete exposure used (e.g., game/practice, minutes of play, season) and how that data was collected	PMID: 25098659, "In men's ice hockey, head impacts for individual players resulting from contact with another player occurred at a frequency of approximately once in every 2 games (0.46 per game)." PMID: 30579266, "The impact rate for each drill was described as the impacts per player per minute (ppm)."	NR
(7f) Outcomes measures, including equations used to derive outcomes, if applicable	Description of each primary and secondary outcome measure (e.g., peak linear acceleration, head impact power)	PMID: 29856659, "Descriptive statistics were calculated for impact counts, locations, and magnitudes in terms of peak linear acceleration (PLA; g), peak angular acceleration (PAA; rad/s2), and HITsp units."	4
(7g) If relevant, equations used to derive outcomes	The algorithms used to derive each outcome measure	PMID: 23864337, "Risks associated with each head impact for each player were summed to compute the risk weighted cumulative exposure (RWE) for the season."	4
(7h) If relevant, categories of direction of impact	Definition and method of determining impact location (e.g., recorded as azimuth and elevation with respect to the head CG)	PMID: 21716150, "Impact location, recorded as azimuth and elevation with respect to the head CG, was categorized into five general location bins (Fig. 2): Front (F), Back (B), Left Side (L), Right Side (R), and Top (T)."	4

27. Arbogast, K.B.; Caccese, J.B.; Buckley, T.A.; McIntosh, A.S.; Henderson, K.; Stemper, B.D.; Solomon, G.; Broglio, S.P.; Funk, J.R.; Crandall, J.R. Consensus Head Acceleration Measurement Practices (CHAMP): Origins, Methods, Transparency and Disclosure. *Ann. Biomed. Eng.* **2022**, *50*, 1317–1345.
